# Knowledge, Experience, and Concerns Regarding Bed Bugs Among Emergency Medical Service Providers

**DOI:** 10.7759/cureus.8120

**Published:** 2020-05-14

**Authors:** Johnathan M Sheele, Osman Hamid, Brandon F Chang, Jeffrey H Luk

**Affiliations:** 1 Emergency Medicine, Mayo Clinic, Jacksonville, USA; 2 Emergency Medicine, University Hospitals Cleveland Medical Center, Cleveland, USA; 3 Emergency Medicine, Case Western Reserve University School of Medicine, Cleveland, USA

**Keywords:** bed bug, bedbug, cimex lectularius, emergency medical service, ems, survey, epidemiology, risk factors, treatment, prehospital

## Abstract

Introduction

Bed bugs are commonly encountered by emergency medical service (EMS) providers. The objective of this study was to determine the frequency with which EMS providers encountered bed bugs, assess their knowledge about bed bugs, and analyze the actions they take after finding bed bugs.

Methods

We anonymously surveyed 407 EMS providers from 180 EMS agencies in northeast Ohio between September 1, 2018, through March 31, 2019.

Results

Among the providers surveyed, 21% (n = 84) of the EMS providers reported seeing bed bugs at least monthly, and 6% (n = 24) reported seeing bed bugs at least weekly. Being younger, male, and working in an urban environment (vs. rural) were associated with EMS providers reporting more frequent bed bug encounters (p: ≤.05). The mean level of concern for encountering bed bugs among EMS providers was 3.54 (SD: 1.15; scale: 1 = no concern, 5 = very concerned). Among the EMS providers who reported seeing bed bugs at least monthly, 30% took the affected EMS stretcher out of service when they encounter a bed bug, 43% took the EMS rig out of service, 83% cleaned the EMS stretcher with a disinfectant, and 88% notified the ED that their patient has bed bugs. EMS providers scored poorly (mean: 69% correct responses) in a seven-question assessment of basic bed bug biology and public health.

Conclusion

Based on our findings, we concluded that EMS knowledge and behavior related to bed bugs are suboptimal.

## Introduction

After near-eradication in the US, *Cimex lectularius* L. (*C. lectularius*), also known as the common bed bug, has been making a resurgence [1,2]. Bed bugs are now one of the most likely ectoparasites encountered by US healthcare providers [3-6]. In a 2018 survey, 59% and 39% of pest management professionals reported that they had decontaminated nursing homes and hospitals for bed bugs, respectively [7]. Emergency department (ED) visits related to bed bugs are estimated to have increased seven-fold between 2007 and 2010 [8].

*C. lectularius* is a hematophagous insect that preferentially feeds on humans [1,9]. The *C. lectularius* life cycle begins with an egg and proceeds through five nymphal stages before becoming an adult [1]. Each life stage requires a blood meal and a molt, and the insect can go from instar to adult in several weeks to months. *C. lectularius* feeds every few days but can go months without a blood meal [1,10]. Adult female *C. lectularius* lays an egg approximately every day when it has access to regular blood meals [1,2]. *C. lectularius* usually seeks refuge near their human host when not seeking a blood meal [1]. Most people develop an itchy rash after being fed upon by a bed bug, and bed bugs can be associated with human anxiety [1,9,11]. *C. lectularius* is not known to be an important vector of human infectious disease, although no major metagenomic investigation has examined its microbiome [1,12-16].

Evidence-based guidelines catering specifically to emergency medical service (EMS) providers on the management of bed bugs do not provide much information apart from generic suggestions about universal precautions, minimizing unnecessary exposure to infested patients, and preventing the spread of the insects to uninfested persons [1,2,17,18]. There have been no studies exploring how EMS providers interact with bed bug-infested patients or assessing EMS provider knowledge about bed bugs. The objective of the study was to determine the frequency with which EMS providers encounter bed bugs, assess their knowledge of bed bugs, and analyze the actions they take after finding bed bugs.

## Materials and methods

Institutional review board approval was obtained from University Hospitals (UH) in Ohio to administer an anonymous survey to approximately 180 EMS agencies under UH medical command in northeast Ohio between September 1, 2018, and March 31, 2019. UH has an existing prehospital bed-bug policy of medical control procedures [19]. We eventually surveyed 407 EMS providers who were all ≥18 years of age. Surveys with incomplete data were included in the final analysis. Only those respondents who reported seeing a bed bug on average every month or more frequently were included in the analyses of the 11 different potential actions that EMS providers could perform when they find a bed bug on a patient.

Continuous variables were analyzed using the t-test and analysis of variance (ANOVA). Categorical variables were evaluated with chi-square analysis. Linear and binomial logistic regression were performed accounting for age, gender (male vs. female), race (white vs. non-white), education (greater than or equal to a bachelor’s degree vs. lower than a bachelor’s degree), hours providing EMS patient care per week (0-20 vs ≥20), level of training [greater than or equal to an emergency medical technician (EMT)-paramedic vs. lower than an EMT-paramedic], the community the EMS provider predominantly serves (urban, rural, or suburban), if they have ever reported having a home bed bug infestation (yes vs. no), ever been fed upon by a bed bug (yes vs. no), ever seen a bed bug (yes vs. no), the number of years doing EMS (≤5, 6-10, 11-15, or >15 years), knowledge of an existing bed bug protocol (yes, no, or unsure), the frequency with which EMS providers ask patients about bed bugs (yes vs. no), and the frequency with which EDs notify the EMS provider that they cared for a patient with bed bugs (yes vs no). Statistical significance was set at a p-value of ≤.05.

## Results

The mean age of the survey respondents was 39.5 years (SD: 12.3 years); 89% (361/404) of the respondents were white, and 93% (376/406) were men. The respondent demographic characteristics and raw responses are summarized in Table 1.

**Table 1:** Demographic characteristics of survey respondents and summary of responses ED: emergency department; EMS: emergency medical services; EMT: emergency medical technician; SD: standard deviation ^a^Values are presented as number (percentage) of patients unless specified otherwise ^b^Percentages do not total 100 because of rounding off

Characteristics	Response^a^ (n = 407)
Age, years, mean (SD)	39.5 (12.3)
Race (n = 404), n (%)	
Non-white	43 (11)
White	361 (89)
Sex (n = 406), n (%)	
Female	30 (7)
Male	376 (93)
Education level (n = 406), n (%)	
Trade/technical/vocational training	126 (31)
Some college credit, no degree	130 (32)
Associate degree	78 (19)
Bachelor’s degree	58 (14)
Master’s or doctorate degree	14 (3)
Highest level of training, n (%)	
First responder	6 (2)
EMT-basic	91 (22)
EMT-intermediate/advanced	8 (2)
EMT-paramedic	302 (74)
Hours/week transporting patients (n = 405), n (%)	
<10	132 (33)
10-20	128 (32)
20-30	61 (15)
30-40	24 (6)
40-50	37 (9)
>50	23 (6)
Most common type of community you serve (n = 406), n (%)	
Rural	67 (17)
Suburban	231 (57)
Urban	108 (27)
EMS experience, years, n (%)	
<1	19 (5)
1-5	66 (16)
6-10	68 (17)
11-15	44 (11)
>15	210 (52)
Frequency with which you see bed bugs while performing EMS duties (n = 406), n (%)	
Never	120 (30)
Less than monthly	202 (50)
Monthly	60 (15)
Weekly	18 (4)
Less than weekly	6 (2)
Ever reported a home bed bug infestation, n (%)	
Yes	16 (4)
No	391 (96)
Ever reported seeing a live bed bug, n (%)	
Yes	270 (66)
No	137 (34)
Ever been fed on by a bed bug (n = 406)^b^, n (%)	
Yes	26 (6)
No	380 (93)
Frequency with which EDs inform you that a bed bug was found on a patient you transported (n = 406), n (%)	
Less than weekly	8 (2)
Monthly	20 (5)
Greater than monthly	169 (42)
Never	209 (52)
Frequency that you ask patients about having bed bugs, n (%)	
Less than weekly	28 (7)
Monthly	24 (6)
Greater than monthly	101 (25)
Never	254 (62)

Frequency of bed bug encounters

Among the survey respondents, 21% (n = 84) of the EMS providers reported seeing bed bugs at least monthly, and 6% (n = 24) reporting seeing bed bugs at least weekly. The EMS providers who reported seeing bed bugs at least weekly primarily worked in urban areas [50% (n = 12)]; while 46% (n = 11) lived in the suburbs, and 4% (n = 1) lived in rural communities. By comparison, the 119 EMS providers who had never seen a bed bug were less likely to work in urban areas [urban: 15% (n = 18); suburban: 56% (n = 67); rural areas: 29% (n = 34); p: <.001]. EMT-paramedics were significantly more likely than non-EMT-paramedics to report seeing bed bugs weekly [83% (20/24) vs. 17% (4/24); p: .002]. EMS providers who see bed bugs at least weekly were significantly more likely to ask their patients about bed bugs and be concerned about personally getting bed bugs (p: <.001 and p: .05, respectively) compared to those EMS providers seeing bed bugs more than weekly. Also, 21% (n = 5) of EMS providers who see bed bugs at least weekly also reported that the ED alerts them that they transported a patient with beds bugs at least weekly on average; while 17% (n =4) reported monthly, 21% (n = 5) reported less than monthly, and 42% (n = 4) never reported at all.

Among the respondents, 80% (86/108) of predominantly urban EMS providers, 66% (152/231) of suburban providers, and 48% (32/67) of rural providers reported previously seeing a bed bug (p: <.001). Regression analysis identified the following EMS variables that were associated with a higher frequency of encountering bed bugs at work: being male, younger age, working in an urban rather than rural community, having previously seen a bed bug, more frequently asking patients about having home bed bugs, and more frequently reporting that an ED notified them that they transported a patient with bed bugs (p: ≤.05 for all) (Table 2).

**Table 2:** Variables associated with the frequency of EMS providers encountering bed bugs EMS: emergency medical services; EMT: emergency medical technician; SE: standard error; CI: confidence interval

Variable	β	t	SE (95% CI)	P-value
Age, years	-0.01	-1.98	0.005 (-0.02 to 0.07)	.05
Sex				
Male vs. female	0.26	2.08	0.12 (0.01 to 0.51)	.04
Race				
White vs. non-white	0.01	0.08	0.13 (-0.24 to 0.26)	.94
Education level				
Greater than or equal to a bachelor’s degree vs. lower than a bachelor’s degree	-0.05	-0.56	0.09 (-0.22 to 0.12)	.58
EMT-paramedic vs. non-EMT-paramedic	0.06	0.69	0.09 (-0.12 to 0.25)	.49
Work in EMS ≥20 vs. 0-20, hours	0.07	0.96	0.07 (-0.07 to 0.21)	.34
Duration of working in EMS, years				
6-10 vs. 11-15	-0.09	-0.67	0.13 (-0.34 to 0.17)	.50
>15 vs. 11-15	0.04	0.30	0.13 (-0.21 to 0.28)	.77
≤5 vs. 11-15	-0.10	-0.75	0.14 (-0.37 to 0.17)	.46
Frequency with which EMS provider asks the patient about bed bugs				
Asks vs. never asks	0.02	5.64	0.04 (0.13 to 0.28)	<.001
Type of community the EMS serves				
Suburban vs. rural	0.02	0.24	0.09 (-0.16 to 0.21)	.81
Urban vs. rural	0.24	1.14	0.11 (0.03 to 0.45)	.03
Ever had a home bed bug infestation				
Yes vs. no	-0.09	-0.47	0.19 (-0.47 to 0.29)	.64
Ever seen a bed bug				
Yes vs. no	0.84	10.68	0.08 (0.68 to 0.99)	<0.001
Frequency with which the ED notifies you that you cared for a patient with bed bugs	0.19	3.68	0.05 (0.09 to 0.30)	<0.001

Concerns about getting bed bugs

The mean level of concern about getting bed bugs among EMS providers was 3.54 (SD: 1.15) (1 = no concern and 5 = very concerned). The mean level of concern about getting bed bugs was 3.8 (SD: 1.2) for providers working predominantly in an urban environment, 3.4 (SD: 1.2) for those working predominantly in a suburban environment, and 3.6 (SD: 1.1) for those predominantly working in a rural environment (p: .02). The variables associated with increased EMS provider concern about getting bed bugs on linear regression were: working ≥20 hours per week in patient care [beta: -0.50 (95% CI: -0.26 to 0.74), p: <.001]; working in a suburban vs. rural environment [beta: -0.34 (95% CI: -0.66 to -0.02), p: 0.04], and asking patients more frequently about possible home bed bug infestations [beta: 0.16 (95% CI: 0.03 to 0.29), p: .02].

Risk factors associated with EMS providers reporting a past home bed bug infestation

Among the respondents, 4% (n = 16) of EMS providers reported that they had a previous home bed bug infestation. Binomial logistic regression identified non-white race [OR: 11.93 (95% CI: 1.97 to 72.33), p: .007] and a previous history of being fed upon by a bed bug [OR: 39.85 (95% CI: 7.58 to 209.41), p: <.001] as risk factors for a home bed bug infestation.

EMS provider knowledge about bed bugs

The mean number of correct responses to seven common bed bug knowledge-based questions was 4.87 (SD: 1.39) with an overall correct response rate of 69% (Table 3).

**Table 3:** Summary of responses to questions relating to knowledge about bed bugs no.: number ^a^Answered correctly

Question	Response, % (no. ratio)
Do bed bugs transmit infectious diseases to humans?	
Yes	39 (157/401)
No	61 (244/401)^a^
Can bed bugs jump?	
Yes	69 (279/406)
No	31 (127/406)^a^
Can bed bugs fly?	
Yes	5 (22/405)
No	95 (383/405)^a^
Do bed bugs feed on human blood?	
Yes	71 (287/405)^a^
No	29 (118/405)
Do bed bugs lay eggs under a person’s skin?	
Yes	25 (102/406)
No	75 (304/406)^a^
Do you think bed bugs live only in unsanitary conditions?	
Yes	18 (72/407)
No	82 (335/407)^a^
Are bed bug infestations easily treated with medication prescribed by a physician?	
Yes	28 (115/405)
No	72 (290/405)^a^
Correct responses, mean	69 (1,970/2,835)

The majority of EMS providers gave correct responses to questions except that 69% (279/406) incorrectly thought bed bugs could jump. Linear regression identified working in a suburban vs. rural environment [beta: 0.46 (95% CI: 0.06 to 0.86)], working in an urban vs. rural environment [beta: 0.56 (95% CI: 0.10 to 1.01)], and working in EMS ≤5 years vs. 11-15 years [beta: -0.64 (95% CI -1.22 to -0.06)] as the variables associated with scoring higher on the bed bug knowledge assessment (p: <.05).

EMS provider actions when encountering bed bugs

The most common actions taken by 21% (n = 84) of EMS providers who reported seeing on average ≥1 bed bug per month was: using extra blankets (58%); inspecting their own clothes for bed bugs (82%); cleaning the EMS stretcher with disinfectant (83%); notifying the ED about the patient having bed bugs (88%) (Table 4).

**Table 4:** Frequency with which EMS providers perform certain actions when they identify bed bugs on patients they are transporting EMS: emergency medical services; ED: emergency department

Action	Responses, n (%)
Do nothing	2 (2)
Use double-glove	10 (12)
Wear face mask	7 (7)
Wear hair nets	4 (5)
Use extra blankets	49 (58)
Inspect own clothes for bed bugs	69 (82)
Clean EMS stretcher with disinfectant	70 (83)
Take EMS stretcher out of service	25 (30)
Steam-clean EMS rig and/or stretcher	18 (21)
Take EMS rig out of service	36 (43)
Notify ED about the bed bug	74 (88)
Hour the EMS rig is taken out of service for a bed bug: <1 hour; 1-8 hours; 9-24 hours; >24 hours	42 (55); 32 (42); 1 (1); 2 (3)

EMS rigs are taken out of service when a bed bug stays on them for more than an hour by 55% of respondents and by 42% when a bed bug stays on them for one to eight hours. Only 2% (n = 2) of EMS providers reported taking no action when seeing bed bugs.

## Discussion

To our knowledge, this is the first study to investigate EMS provider knowledge and behaviors related to bed bugs. Risk factors for EMS providers reporting more frequent exposure to bed bugs included younger age, male gender, and working in an urban environment. EMS providers reported a moderate concern about getting bed bugs (mean: 3.54, on a scale of 1-5), and their knowledge about bed bug public health and biology demonstrated opportunities for more education.

The study population involved EMS agencies in and around Cleveland, Ohio, which is one of the most bed bug-infested cities in the United States [20]. At UH Cleveland Medical Center (UHCMC), a bed bug is found in the facilities on average every 2.2 days and every three to six days in the ED; however, the true number is likely underreported [6,21-23]. A survey of UHCMC ED patients found that about one in 50 reported an active home bed bug infestation; 37% (253/680) reported a past history of being fed on by a bed bug, and 59% (415/702) reported knowing someone other than persons living in their home having a bed bug infestation in the past five years [4,5]. UHCMC ED patients with bed bugs are more likely to be older, male, brought to the ED by EMS providers, and admitted to the hospital [3-5]. Only 18% (n = 2) of the 11 ED patients reporting active home bed bug infestations stated that they notified EMS providers about the infestation [4]. A failure to isolate bed bug-infested patients in the ED puts other healthcare providers and patients at risk for infestation.

Our study has several limitations. Most of our study participants were white, male EMT-paramedics, who worked in a suburban community that has a high number of community bed bug infestations. Most of our respondents have provided EMS services for more than 15 years. The risk to EMS providers for acquiring a home bed bug from work-related duties is difficult to quantify from a survey and remains unknown. EMS provider responses to the knowledge-based questions and their reported actions when caring for bed bug-infested patients demonstrate opportunities for additional education and training.

## Conclusions

EMS providers, especially those working in an urban environment, encounter bed bugs frequently during patient care. Even though EMS providers are concerned about getting bed bugs, they have inadequate knowledge about bed bugs and their effects on human health. Most EMS providers reported low compliance with basic interventions to limit the spread of bed bugs, including taking the EMS rig out of service, cleaning the EMS stretcher with disinfectant, and notifying the ED about a patient having bed bugs.
